# S05-3 How do participants evaluate peer-led walking groups of older adults? Implementation issues related to the ‘Lekker Actief'-project in Flanders

**DOI:** 10.1093/eurpub/ckac093.025

**Published:** 2022-08-29

**Authors:** Filip Boen, Elien Verbaandert, Julie Vanderlinden, Jannique van Uffelen, Jan Seghers, Peter Iserbyt, Katrien Fransen

**Affiliations:** Department of Movement Sciences, KU Leuven, Leuven, Belgium

**Keywords:** physical activity, older persons, implementation, volunteers, participation

## Abstract

Community-based walking groups constitute a promising avenue to increase both the levels of physical activity and the social connectedness of older adults. In addition, while peer-led walking groups seem to be as effective as professional-led programs, they are more cost-effective and socially integrative. In collaboration with a Flemish socio-cultural organization for older adults (i.e., OKRA), we designed the health-promoting program “Lekker Actief” (i.e., “Tastily active”).

This program included an individualized walking scheme for 10 weeks adapted to older adults' fitness level. In addition, in each local meeting point a formal peer leader (i.e., a volunteering older adult who was responsible to implement the guidelines) organized a weekly group walk. Our study involved 19 participating meeting points, counting 504 older adults in total (Mage = 69.2 years) who signed up for the program. In 9 meeting points, peer leadership was distributed among several older adults to create a structure of shared leadership, while in the other 10 meetings points, leadership was assigned to the formal peer leader only.

This presentation will focus on how this (shared) peer leadership was implemented and evaluated. We will draw conclusions to inspire future interventions and policies targeting peer leadership in walking groups for older adults.

